# Ultrafast terahertz saturable absorbers using tailored intersubband polaritons

**DOI:** 10.1038/s41467-020-18004-8

**Published:** 2020-08-27

**Authors:** Jürgen Raab, Francesco P. Mezzapesa, Leonardo Viti, Nils Dessmann, Laura K. Diebel, Lianhe Li, A. Giles Davies, Edmund H. Linfield, Christoph Lange, Rupert Huber, Miriam S. Vitiello

**Affiliations:** 1 Department of Physics, University of Regensburg, 93040 Regensburg, Germany; 2 NEST, CNR-Istituto Nanoscienze and Scuola Normale Superiore, Piazza San Silvestro 12, Pisa, I-56127 Italy; 3 School of Electronic and Electrical Engineering, University of Leeds, Leeds, LS2 9JT UK; 4 Present Address: Fakultät Physik, Technische Universität Dortmund, 44227 Dortmund, Germany

**Keywords:** Quantum cascade lasers, Electronics, photonics and device physics, Polaritons, Terahertz optics

## Abstract

Semiconductor heterostructures have enabled a great variety of applications ranging from GHz electronics to photonic quantum devices. While nonlinearities play a central role for cutting-edge functionality, they require strong field amplitudes owing to the weak light-matter coupling of electronic resonances of naturally occurring materials. Here, we ultrastrongly couple intersubband transitions of semiconductor quantum wells to the photonic mode of a metallic cavity in order to custom-tailor the population and polarization dynamics of intersubband cavity polaritons in the saturation regime. Two-dimensional THz spectroscopy reveals strong subcycle nonlinearities including six-wave mixing and a collapse of light-matter coupling within 900 fs. This collapse bleaches the absorption, at a peak intensity one order of magnitude lower than previous all-integrated approaches and well achievable by state-of-the-art QCLs, as demonstrated by a saturation of the structure under cw-excitation. We complement our data by a quantitative theory. Our results highlight a path towards passively mode-locked QCLs based on polaritonic saturable absorbers in a monolithic single-chip design.

## Abstract (web summary)

Structures that can enhance the capabilities of quantum cascade lasers are highly sought after to improve their practicality for a range of applications. Here the authors demonstrate such a structure in a saturable absorber that takes advantage of intersubband polaritons in the terahertz range and study coherent nonlinear dynamics in the system.

## Introduction

Semiconductor lasers represent a revolutionary milestone in the development of coherent light sources. Laser diodes are compact, energy-efficient and cost-effective devices with extraordinary design flexibility, which has led to countless applications ranging from data storage to ultrafast solid-state lasers^1–3^. While their lasing process exploits interband transitions of electrons, quantum cascade lasers^4,5^ (QCLs) are based on intersubband (ISB) transitions in electrically biased semiconductor multi-quantum well (MQW) structures and extend the emission spectrum towards the mid-infrared and THz spectral regions. Employing sophisticated band structure engineering, their centre wavelength can be tailored with great flexibility while enabling output powers of several watts^6^, as well as large gain bandwidths facilitating frequency comb operation^7–11^ and operation up to room-temperature by intra-cavity difference-frequency mixing^12,13^.

Whereas commercial QCLs available today operate in continuous-wave (cw) mode, there has been a strong demand for QCLs generating few or even single-cycle pulses. Such inexpensive, compact, and highly stable devices could replace complex and expensive table-top laser sources and boost exciting applications ranging from rapid gas sensing via ultrafast communications^6^ to fundamental research investigating low-energy elementary excitations in condensed matter, including superconducting currents^14^, magnons^15^ or Dirac currents^16^, on subcycle time scales. Promising recent demonstrations of short-pulse generation in THz QCLs exploit active mode locking by direct phase synchronization^17^ or by radio frequency modulation of the bias voltage^18–22^, which has enabled record-short pulse durations of 4 ps^21^. Generating single-cycle pulses, however, requires passive mode locking facilitated by saturable absorber (SA) structures with short response times and low saturation thresholds.

**Fig. 1:**
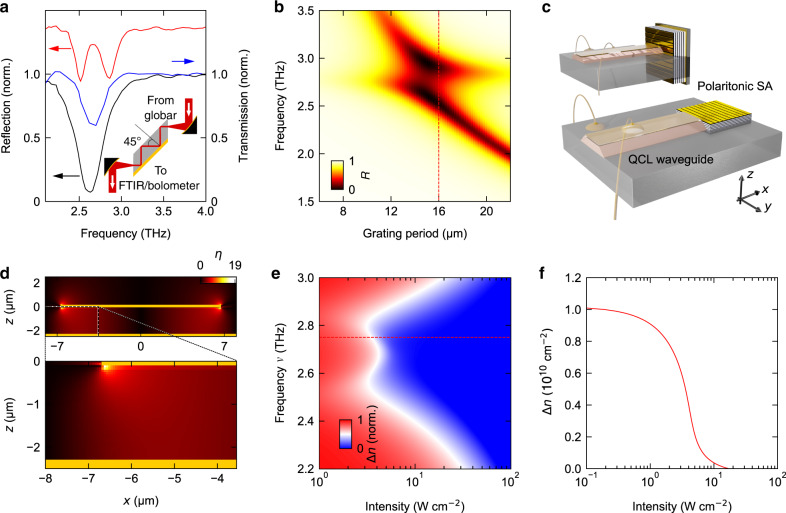
Polaritonic saturable absorber structure. **a** Reflectivity spectra of the coupled structure measured at *T* = 300 K (black curve) and at 6 K (red curve). The blue curve shows the transmission through the QW slab with three internal reflections, measured in a transmission geometry as shown in the inset. **b** Calculated reflectivity *R* as a function of the grating period. The red, dashed line highlights the configuration of our sample with a grating period of 16 µm and polariton resonances located at frequencies of *ν*_LP_ = 2.52 THz and *ν*_UP_ = 2.85 THz, respectively. **c** Schematics of possible options of the QCL design showing the laser structure and the SA replacing one of the laser’s end mirrors. The coordinate systems define the directions *x*, *y* and *z* used throughout the manuscript. **d** *z*-component of the near-field enhancement relative to the incident field amplitude, *η*, within a cross-section of the cavity (top panel), and magnification of the area highlighted by the black rectangle (bottom panel). **e** Population inversion Δ*n* = *n*_1_ − *n*_2_, where *n*_1_ and *n*_2_ are the populations of first and second subband, respectively, as a function of pump intensity and pump frequency, *ν*, calculated using a rate equation model. Red dashed line, ideal pump frequency for saturation. **f** Population inversion Δ*n* as a function of pump intensity, extracted from the data shown in **e**, at 2.75 THz.

While semiconductor-based SA materials are wide-spread in the near-infrared spectral range^23^, only a limited number of concepts exists in the THz domain. Recent demonstrations based on n-doped semiconductors^24^, superconducting metamaterials^25^ or graphene^26,27^ have pioneered this field, yet without achieving simultaneously low saturation intensities and design flexibility regarding the operation wavelength and bandwidth. ISB transitions are promising candidates, enabling straightforward tunability and monolithic integration into the QCL cavity. Still, they suffer from relatively high saturation intensities^28^. A much more powerful solution is provided by ultrastrong coupling of such ISB transitions to the near field of metallic resonators. The field enhancement resulting from the small mode volume of the cavity significantly enhances the optical absorption as compared to the bare electronic transition, where the same value of absorption could only be achieved by an increase of the electron density. Thus, ultrastrong coupling allows reducing the required density of electrons, in turn drastically lowering the threshold of saturation and modifying its dynamics. In this regime of light–matter interaction, the resonator and matter mode periodically exchange energy at a rate known as the vacuum Rabi frequency, Ω_R_, leading to the formation of new eigenmodes called ISB cavity polaritons^29–31^ with a spectral separation of 2Ω_R_. Strong excitation reduces the oscillator strength of the ISB transition and ultimately leads to a collapse of Ω_R_, which may completely bleach the absorption at the resonance frequencies of the polaritons, in equilibrium^32–34^. Owing to the structural similarity of this design and a QCL cavity, a waveguide-type version of this SA could even be integrated into the laser structure and potentially be fabricated in the same epitaxial process (see Fig. 1c). However, it is yet to be determined whether the saturation intensity can be reduced to the level of state-of-the-art QCLs, and whether the onset as well as the decay of saturation are sufficiently fast to allow for few-cycle operation and efficient suppression of cw modes.

In this work, we characterize the coherent ultrafast dynamics of a THz polaritonic SA and reveal a reduction of the saturation threshold by a factor of 9.5 as compared to the bare electronic transition. This significant reduction in saturation intensity is due to the ultrastrong light–matter coupling and, consequently, allows for a 10 times lower electron density than in the weakly coupled case while still maintaining the same strong absorption. We first demonstrate the saturation of the polaritonic system at cw intensities accessible by a THz QCL. Moreover, two-dimensional phase and amplitude-resolved THz strong-field spectroscopy reveals nonlinearities up to six-wave mixing and a dynamical collapse of light–matter coupling, on time scales appropriate for single-cycle operation of a QCL.

## Results

### Design and properties of ISB polaritonic absorbers

We prepare a MQW structure consisting of 35 GaAs quantum wells (QWs) of a thickness of 36 nm and a nominal doping concentration of 5.0 × 10^10^ cm^−2^ charge carriers, per QW. The QWs are separated by Al_0.15_Ga_0.85_As barriers of a thickness of 20 nm. Schrödinger–Poisson calculations predict a dipole moment of *μ*_12_ = 3.8 nm × *e* (*e* is the elementary charge) and an ISB transition frequency of 2.7 THz, which is in good agreement with the value of *ν*_ISB_ = 2.66 THz (Fig. 1a, blue curve) obtained by transmission measurements (Fig. 1a, inset; see also “Methods” section). The cavity consists of a planar gold back surface below the MQW stack and a gold grating structure with a period of 16 µm processed on top (see also Supplementary Fig. 3). A rigorous coupled wave analysis (RCWA) (Fig. 1b) shows that the fundamental cavity mode at a frequency of *ν*_0_ = 2.64 THz couples to the ISB transition, leading to the formation of two distinct polariton states. Reflection spectra of the coupled structure obtained by Fourier transform infrared spectroscopy at a temperature of 10 K reveal the lower (LP) and upper (UP) polariton resonances at frequencies of *ν*_LP_ = 2.52 THz, and *ν*_UP_ = 2.85 THz, respectively (Fig. 1a, red curve), corresponding to a normalized coupling strength of 2Ω_R_/*ω*_0_ = 0.12. The data also allow extracting an effective carrier density of 1.0 × 10^10^ cm^−2^. In contrast, in a room-temperature reference measurement, the thermal population of the second subband state drastically reduces the ISB oscillator strength, leading to a single resonance at the frequency of the fundamental cavity mode (Fig. 1a, black curve). This collapse of the coupling strength should also occur at low temperatures by a strong population of the second subband by THz excitation. FDFD simulations of the ultrastrongly coupled system yield the precise field distribution inside the microcavity, which exhibits regions where the *z*-component of the electric field is enhanced by more than one order of magnitude with respect to the incident electric field (see Fig. 1d). Factoring in the cavity quality factor of 6.7 as well as the oscillator strength and charge carrier concentration of the electronic system, we expect that a fluence of ∼10 W cm^−2^ should realize this situation and lead to a saturation of the polariton resonances^32–34^ (Fig. 1d, e, and see “Methods” section).

### Saturation of ISB polaritons under intense THz-excitation

**Fig. 2:**
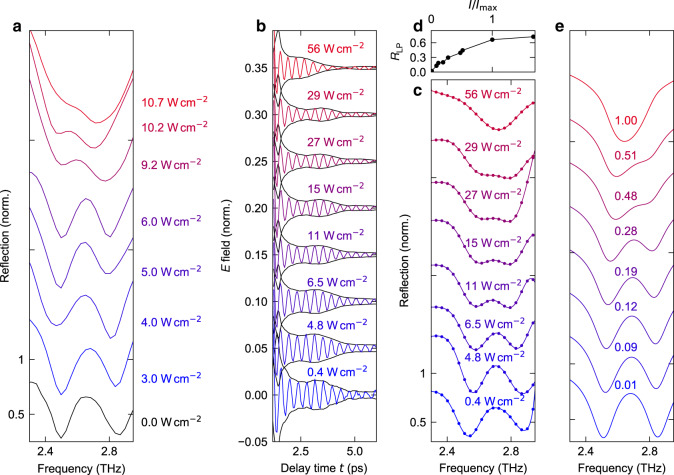
Saturation of reflectivity for high pump intensities. **a** Experimental reflection spectra measured by FTIR during cw excitation at a frequency of 2.75 THz and a peak intensity of up to 11 W cm^−2^. **b** Trailing emitted electric field after excitation with an intense single-cycle THz waveform at a delay time *t* = 0 ps, for various peak intensities. The waveforms are vertically offset for clarity. The envelopes of the electric field are shown as black curves. **c** Corresponding reflectivity spectra, vertically offset for clarity. **d** Reflectivity *R*_LP_ at the frequency of the lower polariton, *ν*_LP_ = 2.53 THz, as a function of the normalized intensity *I*/*I*_max_. **e** Simulated reflection spectra, calculated with a semiclassical approach (see text and “Methods” section) for various peak intensities, given as fractions of the saturation intensity.

We test this idea by probing the reflectivity while exciting the structure with an intense cw THz beam from a QCL under normal incidence, polarized in the *x*–*z*-plane. The pump frequency of 2.75 THz is tuned slightly below *ν*_UP_. This frequency is chosen to account for the red shift expected by saturation and should lead to the lowest saturation intensity. The resulting spectra are plotted in Fig. 2a for various pump intensities, *I*_p_, along with a reference spectrum without excitation (black curve). Whereas for low *I*_p_ (blue curves), the spectral response is only slightly modified, increasing intensities (purple curves) lead to a saturation and a reduction of the polariton splitting, resulting from bleaching of the ISB transition. Eventually, for *I*_p_ = 11 W cm^−2^ (red curve), Ω_R_ is strongly reduced and the polariton resonances partially merge, as expected from the simulation results of Fig. 1e. While a pump-induced reduction of the absorption at the UP resonance already demonstrates the saturation effect required for power modulation in a QCL, the ultrafast polariton dynamics relevant for femtosecond operation remain inaccessible in this cw setting.

Therefore, we proceed by measuring the response to intense single-cycle THz pulses with a variable peak electric field of up to 55 kV cm^−1^ generated by optical rectification of near-infrared laser pulses in a lithium niobate crystal (see “Methods” section). The pulses, polarized in the *x*–*z*-plane, are reflected off the structure under an angle of incidence of 10° and the emitted radiation is detected by electro-optic sampling with subcycle resolution. Figure 2b shows the trailing oscillations of the emitted THz field after the main pulse, whereby the polariton resonances manifest as a pronounced beating feature. Increasing the THz peak field, the beating frequency is slowed down and eventually disappears, resulting in an exponentially decaying monochromatic transient response. The corresponding spectra (Fig. 2c) reveal a progressive reduction of Ω_R_ with increasing pump intensity up to a full collapse of light–matter coupling, and a corresponding increase of the reflectivity of the LP resonance (Fig. 2d), observable even in this scenario of a single pulse acting both as the pump and the probe of the polarization response.

Comparing both scenarios, cw pumping continuously excites carriers slower than the dephasing rate. This condition leads to an incoherent population, bleaching of the transition, and a collapse of Ω_R_. In contrast, strong THz pulses may collapse Ω_R_ on a time scale comparable to the cycle duration of light while preserving the ISB coherence, as shown below. This setting opens up the possibility of energy-conserving pulse shaping, where absorption and subsequent coherent reemission are used to further shorten ultrashort pulses of light^35^. As these coherent dynamics suppress excitation-induced dephasing (EID), a tentatively lower linewidth is obtained as compared to the cw case.

Our semiclassical theory implements excitations of the cavity in the bosonic limit; the dynamics of the electronic excitation are treated by the optical Bloch equations, taking into account the microscopically inhomogeneous near-field profile of our cavity. Light–matter interaction is accounted for including anti-resonant interaction terms beyond the rotating-wave approximation, and the dynamics are solved for the experimental THz waveform (see “Methods” section). Furthermore, we implement EID by a population-dependent polarization decay time *T*_2_, which we retrieve directly from the linewidth of the experimental spectra. The calculation (Fig. 2e) reproduces the experimental spectra and suggests that the inversion of the ISB population and the corresponding collapse of Ω_R_ occur within <1 ps, as inferred from the calculated rise time of the population (not shown). To experimentally access these subcycle nonlinearities we track the coherent and incoherent polarization dynamics directly by a full two-dimensional (2D), field-sensitive THz-spectroscopy.

### 2D THz spectroscopy of ISB polaritons

**Fig. 3:**
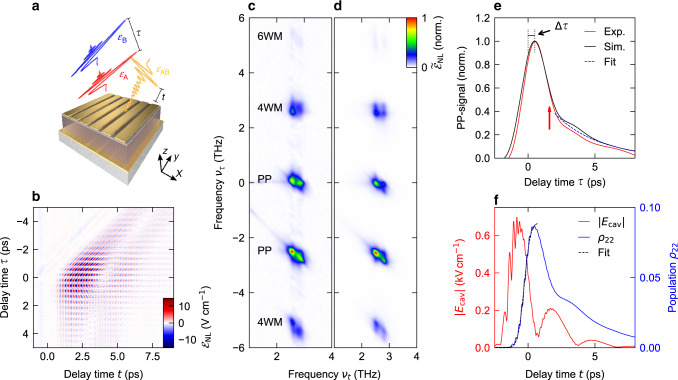
Time-resolved nonlinear dynamics of the polaritonic saturable absorber. **a** Experimental principle of two-dimensional THz spectroscopy. The two THz pulses (red and blue waveforms, delayed by a delay time *τ*) prepare and probe the nonlinear dynamics of the saturable absorber. The emitted THz waveform ${\mathcal{E}}$_AB_ is sampled electro-optically as a function of the delay time *t*. **b** Colour plot of the emitted nonlinear electric field, ${\mathcal{E}}$_NL_ = ${\mathcal{E}}$_AB_ − ${\mathcal{E}}$_A_ − ${\mathcal{E}}$_B_ as a function of the electro-optic delay time *t* and *τ* at a peak THz intensity of *I* = 7 W cm^−2^. **c** Two-dimensional Fourier transform of the electric field in **b**. **d** Simulated 2D spectra, calculated from a semiclassical theory. **e** Experimental (red curve) and simulated pump–probe signal (black curve) as a function of the delay time *τ* for a fixed electro-optic delay time of *t* = 1.66 ps. The maxima are offset from time zero by Δ*τ* = 0.5 ps. Dashed blue curve: exponential decay with a characteristic time of 3.3 ps, as a guide to the eye. **f** Calculated dynamics of the cavity field amplitude (|*E*_cav_|, red curve), and the population of the second subband (*ρ*_22_, blue curve). Black dashed curve, fit of *ρ*_22_ by a time-integral of a Gaussian envelope function, demonstrating a rise time of ∼900 ps.

To this end, two phase-locked, collinearly propagating THz pulses (labelled A and B), which are polarized in the *x*–*z*-plane, are delayed by a variable time *τ* and focused onto the structure. The reflected waveform ${\mathcal{E}}$_AB_ is detected by electro-optic sampling (Fig. 3a). We modulate both beams independently of each other and subtract their individual fields ${\mathcal{E}}$_A_ and ${\mathcal{E}}$_B_ from ${\mathcal{E}}$_AB_, which allows us to extract the emitted field ${\mathcal{E}}$_NL_ resulting from nonlinear multi-photon interaction processes. By varying *τ*, we control the corresponding phases of the individual pulses. Figure 3b shows ${\mathcal{E}}$_NL_ as a function of *t* and *τ*, for a THz peak intensity of 7 W cm^−2^. Fronts of constant phase of pulse A appear as vertical lines, while those of pulse B are slanted by 45°, since its field crests are located at constant values of *t*–*τ*. Within the temporal overlap region of the polarization induced by the pulses (simultaneously *t* > 0 ps and *t* + *τ* > 0 ps), ${\mathcal{E}}$_NL_ is coherently modulated along both temporal axes, whereby ${\mathcal{E}}$_NL_ is strongest for a temporal coincidence of both pulses, at *τ* = 0. Here, an interference pattern around *t* ≈ 4 ps attests to a beating of the nonlinear polarizations of the two polariton resonances.

More quantitatively, we disentangle the nonlinear response into its contributions by a two-dimensional Fourier transform, and plot the spectral amplitude $\tilde{\mathcal{E}}_{{\mathrm{NL}}}$ as a function of the frequencies *ν*_*t*_ and *ν*_*τ*_ associated with *t* and *τ*, respectively (Fig. 3c). The spectra reveal five groups of nonlinear processes including pump–probe (PP) interactions resulting from incoherent saturation of the ISB transition, as well as four-wave (4WM) and six-wave mixing (6WM) contributions associated with nonlinear polarization dynamics, where each group features distinct narrowband signatures of both polariton branches (see “Methods” section for the polarization underlying different nonlinearities). We again support this analysis by our microscopic theory, on a quantitative level (Fig. 3d).

Next, we extract the dynamics of the PP signal near *ν*_*τ*_ = 0 THz by bandpass filtering of the corresponding spectral range and back-transformation into the time domain, which reveals the decay as a function of *τ* for a fixed delay time *t* = 1.66 ps (Fig. 3e, red curve). Initially, the amplitude follows the temporal shape of the incident pulses, thus including a rapid initial increase and subsequent decay. The slight delay of the maximum at Δ*τ* = 0.5 ps is associated with the time required to transfer energy from the cavity to the electronic excitation. From *τ* = 2.5 ps onward, pulse A interacts with the residual polarization induced by pulse B and thus directly monitors the population dynamics. Owing to the periodic energy exchange between cavity and electronic system, the net decay rate alternates between the faster decay of the cavity (black arrow), and the slower decay of the electronic excitation. Fitting the data in this delay range with an exponential function, we find an averaged characteristic population decay time of *T*_1_ = 3.3 ps.

This dynamics is well confirmed by our simulations (Fig. 3f). The modulus of the cavity field (red curve) rises steeply upon excitation by the external THz pulse before the energy is almost completely transferred to the two-level system, within a time delay set by half of the vacuum Rabi cycle. The strong PP signal emerges in lock with the population dynamics. Fitting the steep rise of the population for *t* < 0 ps with the time-integral of a Gaussian envelope function, we extract a FWHM of the excitation time of only 900 fs (broken curve in Fig. 3f). For the weak external driving shown here, the energy contained in the ISB system is subsequently fed back into the cavity while the population rapidly decreases, manifesting in the slight modulation of the PP signal for *τ* > 0 seen in Fig. 3e. In case of a strong excitation, however, population inversion is reached and the strong coupling breaks down, leading to a saturated absorption (see Supplementary Fig. 2). These ultrafast response and recovery dynamics are ideally suited for pulsed few-cycle operation of a QCL with pulse durations as short as two picoseconds, only limited by the excitation time of the SA. We note that the achievable pulse duration may be yet shorter, as even a partial saturation may provide the required preference of the pulsed operation over the cw mode.

### Saturation intensity of ISB polaritons

**Fig. 4:**
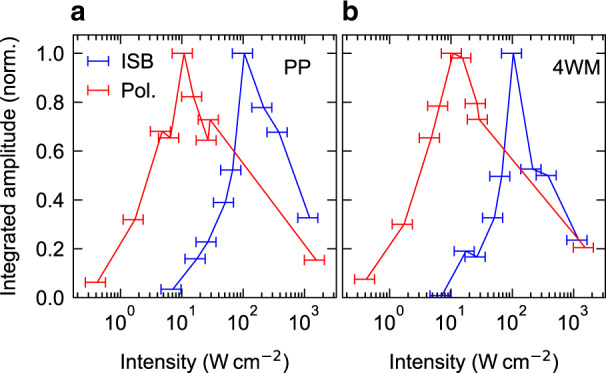
Dynamics of the nonlinear response as a function of pump intensity. **a** Integrated PP signal (red curve) as a function of peak intensity of the incident THz pulses. The blue line indicates the experimental values for a bare ISB transition. The error bars state the systematic uncertainty of the determination of the peak intensity. **b** Corresponding curves for the 4WM signal.

Beyond these favourable ultrafast characteristics, the ultrastrong light–matter coupling of our polaritonic system promises significantly reduced saturation intensities as compared to the bare electronic transition^28^. From a systematic variation of the incident THz field amplitude, we find that the amplitudes of the PP (Fig. 4a, red curve) and 4WM signals (Fig. 4b, red curve) initially increase with increasing pump intensity until both reach a distinct maximum at an intensity of 10 W cm^−2^. A further increase of the pump intensity saturates the ISB transition, which reduces the strength of light–matter coupling and leads to a reduction of the nonlinear signal. In comparison, the nonlinearities of the bare ISB transition^28^ show qualitatively similar behaviour (Fig. 4a, b, blue curves), yet for a tenfold increase of the intensity. This significant scaling advantage of our polaritonic approach allows saturation intensities well achievable in state-of-the-art QCLs, as demonstrated by our findings in the cw experiments. Moreover, the saturation intensity under pulsed excitation, corresponding to a fluence of 0.1 nJ cm^−2^, is several orders of magnitude lower than in previous demonstrations, where up to several tens of µJ cm^−2^ were required^24,25^, and comparable to graphene-based approaches^26,27^. Our structure’s non-saturable losses of only 15% are significantly lower than previously reported values ranging between 40% (ref. ^25^) and 85% (ref. ^24^), while the reflectivity doubles under saturation, representing a high energy efficiency relevant for low-power devices.

## Discussion

In conclusion, we have introduced an ultrafast SA implemented by THz ISB polaritons in a semiconductor structure suitable as a monolithically integrated intra-cavity element of a THz QCL. Strong-field excitation by single-cycle THz waveforms has unveiled coherent nonlinear dynamics including up to six-wave mixing processes, which lead to a transient collapse of light–matter coupling on subcycle time scales. The resulting strong modulation of the optical response within a single cycle of the carrier wave and the recovery time as short as 3.3 ps open the door to passive, self-starting mode-locking of QCLs emitting few-cycle THz pulses. Increasing the light–matter coupling even further by improving the field confinement or increasing the dipole moment of the ISB transition may enable even faster saturation dynamics and larger vacuum Rabi splitting, increasing the operational bandwidth and response times towards single-cycle operation at high repetition rates. Moreover, the large degree of coherence offers the prospect for energy-conserving, lossless pulse shaping. Specifically, four-wave and six-wave-mixing nonlinearities discovered here, may lead to drastic efficiency improvements over designs relying solely on incoherent absorption. Our concept provides quantitative design guidelines for a new generation of compact, cost-effective, electrically driven sources of intense THz pulses for a broad range of applications in ultrafast sciences, data storage, high-speed communication and spectroscopy.

## Methods

### Device fabrication

The polaritonic absorbers were fabricated by evaporating a gold layer of a thickness of 500 nm onto the QW sample, and an n^++^-GaAs substrate, both of a thickness of 500 µm. In a next step, the two parts were joined by thermo-compressive Au–Au wafer bonding. Subsequently, the substrate of the QW sample was etched down to a thickness of 2.03 μm, first via mechanical polishing and then by wet-chemical etching, until the etch-stop layer composed of Al_0.5_Ga_0.5_As was reached. The latter was then selectively removed in a second wet-etching chemical process. A one-dimensional photonic structure based on a supercell scheme including a number of *N*_Bragg_ identical gold stripes (periodicity, 16 µm) was fabricated on the top surface of the MQW stack using a two-step optical lithographic process, followed by thermal evaporation of a Cr/Au layer (thicknesses, 10 nm/100 nm) and lift-off. Finally, the processed samples were mounted on a copper sub-mount for reflection/transmission measurements at cryogenic temperatures.

### Fourier-transform infrared (FTIR) spectroscopy

The polaritonic samples were characterized by FTIR spectroscopy employing an evacuated spectrometer (Bruker, Vertex 80), a broadband globar source, and a silicon bolometer. Multi-pass transmission experiments (Fig. 1a, inset) were performed for the bare MQW sample after lapping its front and back facets at an angle of 45°. Reflectance measurements were carried out for an angle of incidence of 10° for various temperatures of the heat sink ranging from room temperature (RT) down to 6 K. The small angle of 10° minimizes frequency shifts of the resonator, compared to the resonance at normal incidence. For sufficiently low temperatures, the upper photonic mode strongly couples with the ISB transition, leading to the formation of the lower and upper polariton modes with a frequency splitting of 0.34 THz. Pump–probe experiments were performed by exciting the structure with the cw QCL (see Supplementary Fig. 5 for emission spectra) operating at a frequency of 2.75 THz, under normal incidence, while probing the reflectivity under an angle of 10°. The entire measurement setup was housed in an evacuated chamber.

### Calculation of steady-state saturation

We quantitatively calculate the saturation threshold for cw excitation using a frequency-dependent rate-equation model. The rate of absorption takes the form

1 $$R\left( {{\mathrm{{\Delta} }}n,\omega } \right) = n_2\frac{{\hbar \omega }}{{\tau _{{\mathrm{ISB}}}I}},$$

where *n*_2_ is the charge density in the second subband, *τ*_ISB_ is the ISB relaxation time, *I* is the incident intensity, and *R* is the reflectance, which depends on the frequency *ω* and the difference of the populations of the first and second subbands, Δ*n* = *n*_1_ − *n*_2_. The conservation of charge carriers, *n*_1_ + *n*_2_ = *n*(0), where *n*(0) is the chemical doping density, allows us to solve Eq. (1) for *n*_2_, leading to the density of excited carriers as a function of intensity, Δ*n*(*I*) as shown in Fig. 1e. Moreover, we apply a coupled-mode theory to calculate the reflectivity spectra, taking into account the reduction of the coupling strength Ω_R_ as a result of the saturation of the ISB transition, leading to

2 $$R\left( {{\Delta} n,\omega } \right) = \frac{{2\gamma _{\mathrm{c}}\gamma _{{\mathrm{ISB}}}{\Omega} _{\mathrm{R}}^2}}{{\left( {\omega - \omega _0} \right)^4 + \left( {\omega - \omega _0} \right)^2\left( {\gamma _{\mathrm{c}}^2 + \gamma _{{\mathrm{ISB}}}^2 - 2{\Omega} _{\mathrm{R}}^2} \right) + \left( {{\Omega} _{\mathrm{R}}^2 + \gamma _{\mathrm{c}}\gamma _{{\mathrm{ISB}}}} \right)^2}},$$

where the coupling strength Ω_R_ is

3 $${\mathrm{{\Omega} }}_{\mathrm{R}} = {\xi}\sqrt {\frac{{\pi e^2{\mathrm{{\Delta} }}n}}{{{\it{\epsilon }}_wm\ast L_{{\mathrm{MQW}}}}}}.$$

Here, ${\it{\epsilon }}_w$ is the permittivity of the quantum well material, m* is the effective mass of the conduction band electron, *L*_MQW_ is the thickness of the MQW stack, and *ξ* represents the field overlap factor, i.e. the overlap of light and matter modes, calculated via the RCWA method. *γ*_c_ and $\gamma _{{\mathrm{ISB}}} = {\textstyle{1 \over {\tau _{{\mathrm{ISB}}}}}}$ denote the damping constants of the cavity and the ISB system, respectively.

In Eq. (2), *ω*−*ω*_0_ is the detuning of the photon energy from the cavity resonance. The analytic expression of Eq. (2), plugged into Eq. (1), justifies the saturation behaviour observed in Fig. 1e. Indeed, when a narrow-band excitation at a frequency *ω*_0_ is considered, the function *R*(Δ*n*, *ω* = *ω*_0_) has to be employed. This function is not monotonic with respect to Ω_R_ (and, consequently to Δ*n*), hence triggering the observed sharp saturation.

### 2D THz spectroscopy

Carrier-envelope-phase-stable, single-cycle THz pulses (Supplementary Fig. 1) are generated by optical rectification of near-infrared pulses of a duration of 260 fs from an Yb:KGW amplifier in a cryogenically cooled (77 K) lithium niobate crystal in a tilted pulse-front scheme^36^. We use a Michelson interferometer with a silicon beamsplitter to separate the generated THz pulses into two identical pulses, delayed with respect to each other by a variable delay time *τ*. After reflection from the sample, the total THz electric field is detected by electro-optic sampling in a GaP crystal of a thickness of 1 mm with a small portion of the near-infrared light used as a gate pulse.

The origin of the nonlinear pump–probe and four-wave mixing signals from the sample can be understood in terms of third-order nonlinear polarizations. For example, the pump–probe signals at *ν*_*τ*_ = 0 THz originate from polarization components

4 $$P_{{\mathrm{PP}}}^{\left( 3 \right)}\left( \nu \right) = \chi ^{(3)}\left( {\nu ,\nu , - \nu } \right)\tilde{\mathcal{E}} _{\mathrm{A}}\left( \nu \right)\tilde{\mathcal{E}} _{\mathrm{B}}\left( \nu \right)\tilde{\mathcal{E}} _{\mathrm{B}}^ \ast \left( { - \nu } \right),$$

whereby *ν* represents the absorption of a photon, while −*ν* represents the emission of a photon into the respective field component. In this particular case, one photon is absorbed from field A while for field B, one photon is absorbed and a second one is emitted, such that the process is incoherent with respect to the phase of field B and called a pump–probe process. In contrast, a coherent four-wave mixing nonlinearity as

5 $$P_{4{\mathrm{WM}}}^{\left( 3 \right)}\left( \nu \right) = \chi ^{\left( 3 \right)}\left( {\nu ,\nu , - \nu } \right)\tilde{\mathcal{E}} _{\mathrm{A}}\left( \nu \right)\tilde{\mathcal{E}}_{\mathrm{A}}\left( \nu \right)\tilde{\mathcal{E}}_{\mathrm{B}}^ \ast \left( { - \nu } \right),$$

preserves the phases of both fields, A and B. In conclusion, pump–probe processes are a measure of the incoherent population of the ISB system, while four-wave mixing processes represent the coherent nonlinear polarization. Moreover, a convenient graphical representation is obtained when the 2D frequency plots of Fig. 3c, d (see also Supplementary Fig. 4) are traversed along a path of wave vectors, called a Liouville path. Each vector represents one photon of the incident fields contributing to the above third-order nonlinear polarization^37^. Correspondingly, each location (*ν*_*t*_, *ν*_*τ*_) in 2D frequency space can be reached by an integer linear combination of the wave vectors **k**_A_ = (*ν*_ISB_, 0) and **k**_B_ = (*ν*_ISB_, −*ν*_ISB_) with non-zero coefficients. For example, the signals at **k**_PP1_ = (*ν*_ISB_, 0) and **k**_PP2_ = (*ν*_ISB_, −*ν*_ISB_) are represented by Liouville paths **k**_PP1_ = **k**_A_ + **k**_B_ − **k**_B_ and **k**_PP2_ = **k**_A_ − **k**_A_ + **k**_B_, respectively. As in these cases, the phase of either pulse B (for **k**_PP1_) or pulse A (for **k**_PP2_) cancels, the processes describe pump–probe interactions. In contrast, the coherent four-wave mixing signals at **k**_4WM1_ = (*ν*_ISB_, *ν*_ISB_) and **k**_4WM2_ = (*ν*_ISB_, −2*ν*_ISB_) can be expanded as **k**_4WM1_ = **k**_A_ + **k**_A_ − **k**_B_ and **k**_4WM2_ = **k**_B_ + **k**_B_ − **k**_A_, conserving the phases of both fields.

### Microscopic calculation of polaritonic dynamics

We model the dynamic response of our structure under intense THz excitation by a semiclassical theory of ultrastrong light–matter interaction. The Hamiltonian reads^38^

6 $$\hat H = \hat H_{{\mathrm{ISB}}} + \hat H_{{\mathrm{cav}}} + \hat H_{{\mathop{\rm{int}}} } + \hat H_{{\mathrm{ext}}}.$$

Here, $\hat H_{{\mathrm{ISB}}} = \hbar \omega _{{\mathrm{ISB}}}\hat b^\dagger \hat b$, and $\hat H_{{\mathrm{cav}}} = \hbar \omega _{{\mathrm{cav}}}\hat a^\dagger \hat a$ account for the ISB polarization field and the cavity field, respectively. $\hat b$ and $\hat a$ denote the corresponding annihilation operators. Light–matter interaction is implemented including anti-resonant interaction terms, leading to $\hat H_{{\mathrm{int}}} = \hbar {{\mathrm{\Omega} }_{\mathrm{R}}}( {\hat b + \hat b^\dagger } )\left( {\hat a + \hat a^\dagger } \right) + \hbar D\left( {\hat a + \hat a^\dagger } \right)^2$, where Ω_R_ is the vacuum Rabi frequency. While anti-resonant contributions may be neglected for low coupling strengths 2Ω_R_/*ω*_0_ << 1 and weak excitation, here, we model a situation of 2Ω_R_/*ω*_0_ = 0.12 and strong excitation by single-cycle pulses with peak electric fields of several kV cm^−1^, which requires these light–matter interaction terms to be included. We note that the diamagnetic shift of the cavity, $D = {\Omega}_{\mathrm{R}}^2/\omega _0$, is small in our case and could be neglected, in principle. However, since it originates from the same formalism as the anti-resonant light–matter interaction terms and would have to be included for the discussed scenarios of larger coupling strengths, we have kept it in the theory. The excitation of the cavity mode by the external field is contained in $\hat H_{e{\mathrm{xt}}}$. We calculate the dynamics of the cavity and polarization fields using Heisenberg’s equation of motion in the mean-field limit, where $\alpha (t) = \langle \hat a(t) \rangle$ and $\beta (t) = \langle \hat b(t) \rangle$. We obtain the equation of motion for *α*

7 $$\dot \alpha = - i\omega _{{\mathrm{cav}}}\alpha - \gamma _c\alpha - 2iD(\alpha + \alpha \ast ) - i{\Omega}_{\mathrm{R}} \left( {\beta + \beta \ast } \right) + {\mathcal{E}}\left( t \right),$$

where we introduced the phenomenological damping constant *γ*_c_ of the resonator. The driving term ${\mathcal{E}}$(*t*) represents the external electric field of the incident THz transient.

The ISB transition is modelled as an ideal two-level system, which can be well described in terms of a density matrix $$\rho = \left( {\begin{array}{*{20}{c}} {\rho _{11}} & {\rho _{12}} \\ {\rho _{21}} & {\rho _{22}} \end{array}} \right)$$, where *ρ*_11_ (*ρ*_22_) is the population of the first (second) subband, and $\rho _{12} = \rho _{21}^ \ast$ is the ISB polarization. Applying the von Neumann equation we calculate the time derivatives of the individual matrix elements as

8 $$\begin{array}{l}\dot \rho _{11} 	= i{\mathrm{{\Omega}}_{\mathrm{R}}}\left( {\alpha + \alpha \ast } \right)\left( {\rho _{21} - \rho _{12}} \right) + \dot \rho _{22}/T_1, \hfill\\ \dot \rho_{22} 	= - \dot \rho_{11},\hfill\end{array}$$

9 $$\dot \rho _{12} = - i\omega _0\rho _{12} - \gamma _{{\mathrm{ISB}}}\rho _{12} - i{\mathrm{\Omega}}_{\mathrm{R}}(\alpha + \alpha \ast )\left( {\rho _{22} - \rho _{11}} \right).$$

These coupled differential equations are solved numerically, where the constants *ω*_0_, *γ*_ISB_, *γ*_c_, and Ω_R_ are determined from the linear characterization of the sample. The reflection spectra in Fig. 2c are then calculated by a superposition of the incident electric field ${\mathcal{E}}$(*t*) and the cavity field *α*(*t*). The nonlinear response *α*_NL_(*t*, *τ*) is determined by calculating the response *α*_AB_(*t*, *τ*) for a driving field ${\mathcal{E}}$_AB_(*t*, *τ*) = ${\mathcal{E}}$_A_(*t*) + ${\mathcal{E}}$_B_(*t* − *τ*) and subtracting the individual contributions *α*(*t*) and *α*(*t*, *τ*) for driving fields ${\mathcal{E}}$_A_(*t*, *τ*) = ${\mathcal{E}}$_A_(*t*) and ${\mathcal{E}}$_B_(*t*, *τ*) = ${\mathcal{E}}$_B_(*t* − *t*), respectively.

### Polariton dynamics in the high-field regime

In contrast to weak excitation of the sample (see Fig. 3f and “Discussion” section), an increase of the electric peak field by a factor of five leads to an almost complete bleaching of the ISB transition, i.e., a population inversion Δ*n* = *n*_2_ − *n*_1_ ≈ 0. Consequently, the strong light–matter coupling breaks down, which manifests itself in a monotonic decrease of both the cavity field as well as the population, governed by the coupling to the far field (see Supplementary Fig. 2).

## Supplementary information

Supplementary Info

## Data Availability

The data that support the plots within this paper and other findings of this study are available from the corresponding authors upon reasonable request.
