# Supplementary material for: Ultrafast terahertz saturable absorbers using tailored intersubband polaritons
Source: Nat Commun. 2020 Aug 27;11:4290. doi: 10.1038/s41467-020-18004-8 (PMC7453201; doi:10.1038/s41467-020-18004-8)
Supplement: Supplementary file 1 — Supplementary Info [file 41467_2020_18004_MOESM1_ESM.pdf]

Supplementary Information for

"Ultrafast terahertz saturable absorbers using tailored intersubband polaritons"

J. Raab et al.

Supplementary Figures

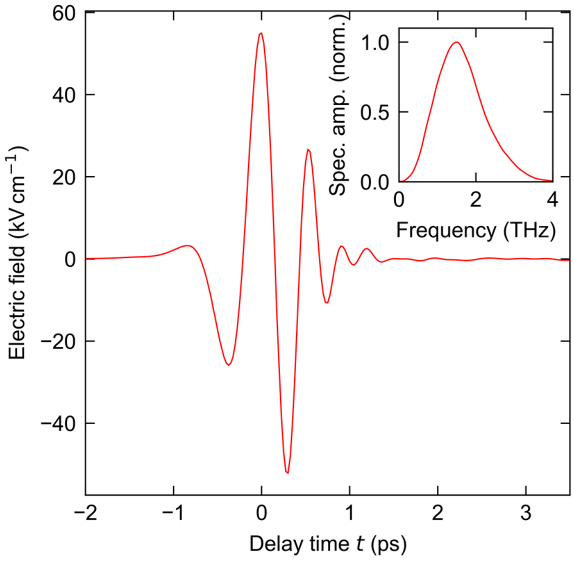

Supplementary Figure 1. Experimental THz waveform. THz electric field of the employed THz transient as a function of the delay time t, measured by electro-optic detection. Inset: Fourier spectrum of the THz transient.

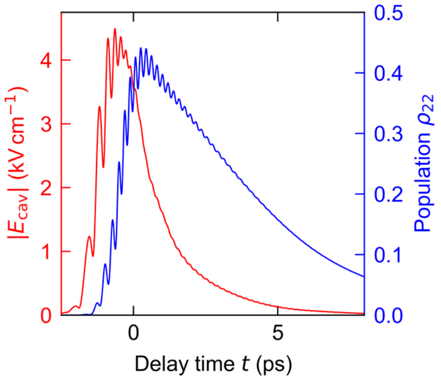

Supplementary Figure 2. Polariton dynamics in the high-field regime. Calculated dynamics of the cavity field amplitude (red curve), and the population of the second subband (blue curve) for an excitation with an electric field five times stronger than in Fig. 3f.

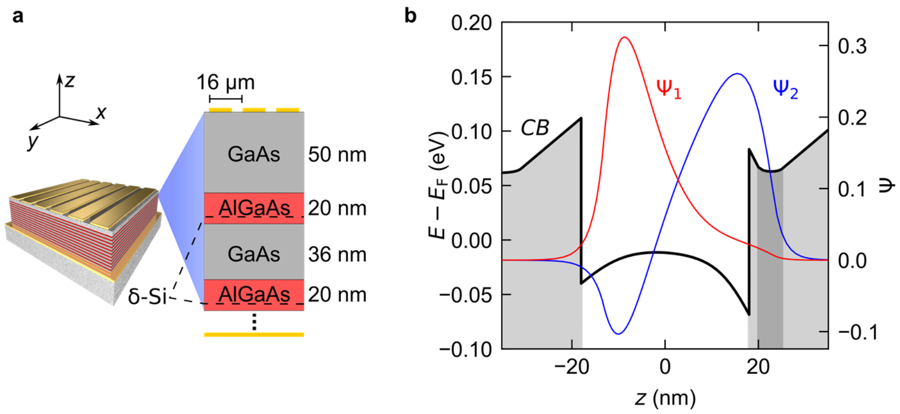

Supplementary Figure 3. Material parameter of the saturable absorber. a, Schematic of the multi-quantum well stack as well as the gold grating and the back metallization layer. δ-Si: delta-Silicon doping. **b**, Simulated, conduction band edge (CB, black) of the MQW structure. Ψ_1 and Ψ_2 are the electron envelope functions of the first (red) and second (blue) subband.

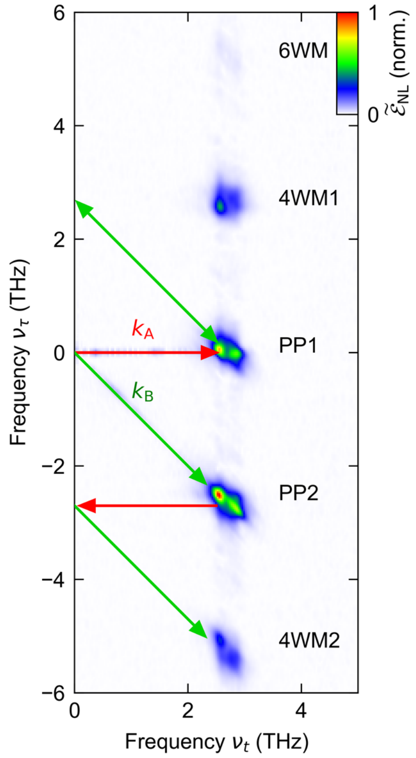

Supplementary Figure 4. Liouville path analysis of 2D Frequency map. 2D Fourier transform of the time domain signal in Fig. 3b, revealing pump-probe (PP1, PP2) and four-wave mixing (4WM1, 4WM2) signals. The Liouville paths corresponding to PP1 and 4WM2 are represented by their respective superposition of wave vectors of the fields $\mathcal{E}_A$ ($\mathbf{k}_A$, red arrows) and $\mathcal{E}_B$ ($\mathbf{k}_B$, green arrows).

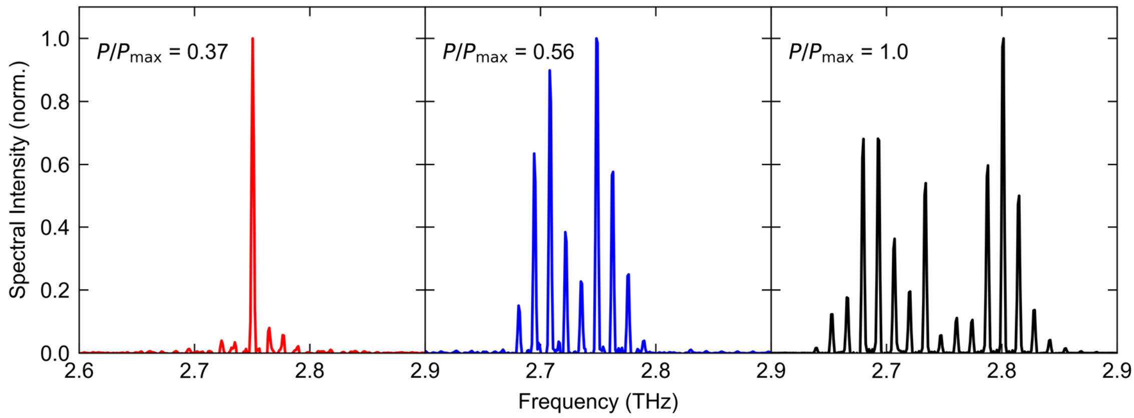

Supplementary Figure 5. QCL spectra. Intensity spectra of the QCL used for pumping the SA structure for various powers P. P_{max} corresponds to the power at the maximum intensity incident on the sample in Fig. 2a.
